# Selecting the Appropriate Radiation Therapy Technique for Malignant Spinal Cord Compression Syndrome

**DOI:** 10.3389/fonc.2019.00065

**Published:** 2019-03-04

**Authors:** Jun-Ho Lee, Seok Ho Lee

**Affiliations:** ^1^Department of Emergency Medical Technology, Daejeon University, Daejeon, South Korea; ^2^Department of Radiation Oncology, Gil Medical Center, Gachon University of Medicine and Science, Incheon, South Korea

**Keywords:** radiation therapy (radiotherapy), spinal cord compression, intensity modulation radiation therapy (IMRT), cancer, emergency

One of the most common metastatic lesions in cancer patients is bone metastasis. Spinal metastasis, in particular, is a more noticeable form of bone metastasis due to the symptoms of spinal metastasis and the urgency of treatment. Manifestations of the symptoms that require such emergency treatment are caused by spinal metastasis that leads to spinal cord compression syndrome. Spinal cord compression syndrome is caused by the compression of the spinal cord, especially that of the epidural spinal cord by a tumor. The most common causes of spinal cord compression syndrome due to bone metastasis are lung cancer and breast cancer. The most common involvement site is the thoracic region; the most common symptom is pain (1).

Spinal metastasis may cause pain, as well as induce neurological damage, which can then lead to a dramatic deterioration in a patient's quality of life due to sensory changes, a loss of strength, a loss of rectal capacity, and intestinal obstruction. Spinal metastasis is a representative disease requiring emergency treatment (2).

Steroids should be given as a first treatment upon diagnosis, and surgery or radiation therapy (RT) should be performed depending on the extent of the compression of the spinal cord (3). There is a question as to whether surgery or SBRT should be performed first on patients with malignant spinal cord compression syndrome. According to Ryu et al. (4), in the case of a grade IV or V epidural compression, urgent treatment for the spinal cord compression is especially important, and surgery should be considered. If surgery is not possible, RT is needed.

It is of primary importance for patients and caregivers to be aware of pain, which is the initial symptom of spinal cord compression syndrome and to promptly notify the primary care physician when symptoms develop (5).

Three-dimensional conformal radiation therapy (3D-CRT) has been considered the traditional standard of practice to treat spinal metastasis. In general, the main goal of 3D-CRT is to deliver a conformal dose distribution to the target volume, reducing the radiation dose to the normal tissues (Figure 1). Although, a conformal treatment plan for the target volume using 3D-CRT planning can be developed, minimizing the radiation doses to the critical spinal cord area is generally not possible, which prevents it from delivering the maximum radiation doses to the target volume.

**Figure 1:**
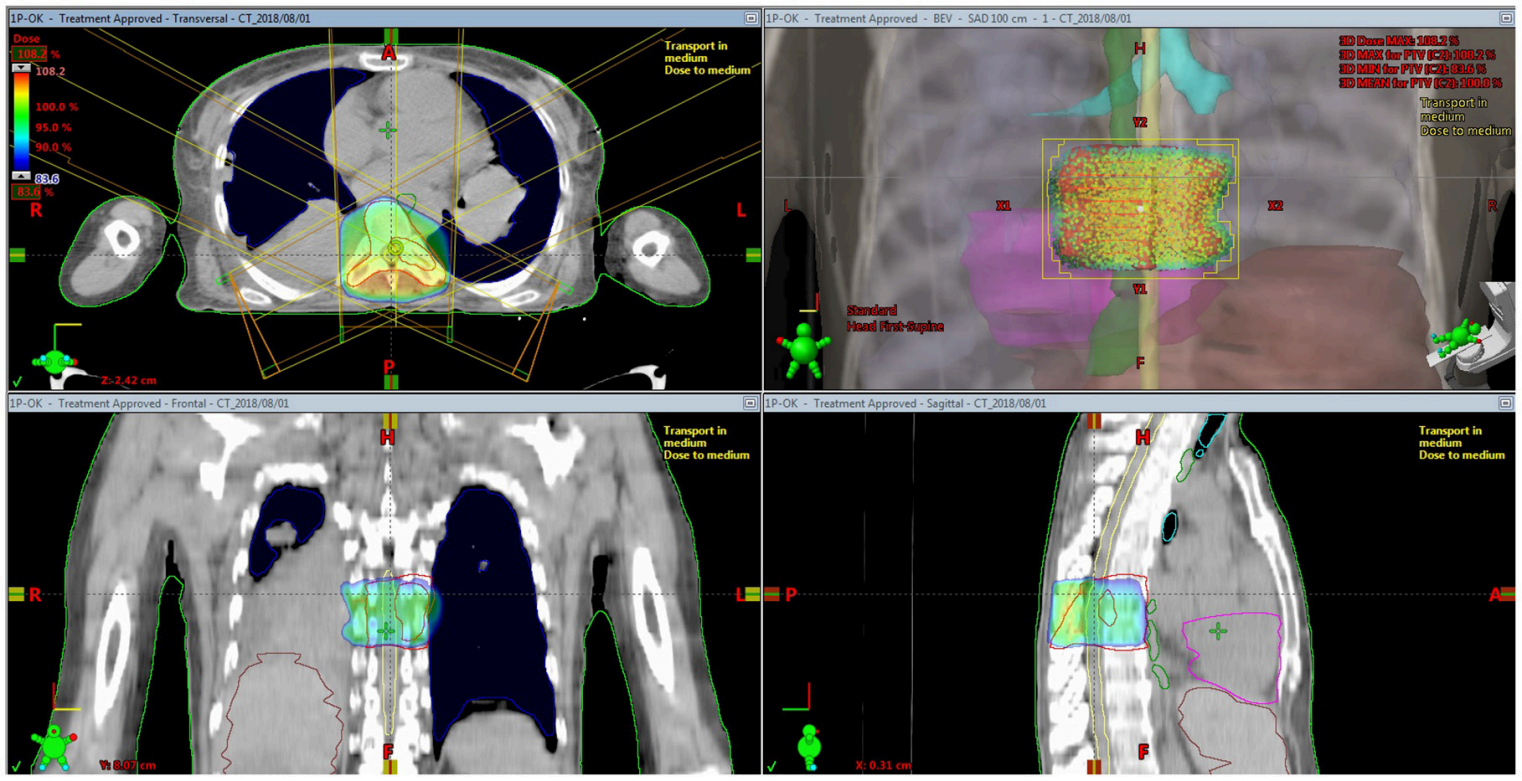
Isodose distribution for three-dimensional conformal radiation therapy for spine metastasis.

Recently, intensity-modulated radiation therapy (IMRT) has been introduced to treat spinal metastasis. IMRT can make it possible to safely deliver the optimal radiation doses to irregularly shaped tumors. Furthermore, the use of IMRT, which has excellent efficacy in protecting the normal tissue, especially the spinal cord, during treatment, also increases the probability of tumor control; an improved treatment response including complete remission, which could not have been expected from conventional 3D-CRT, can be attained. By using these IMRT techniques, stereotactic body radiation therapy (SBRT) has been widely adapted for the treatment of patients with spinal metastasis.

SBRT was defined as “the precise delivery of highly conformal and image guided hypo-fractionated external beam radiation therapy (IGRT), delivered in a single or few fractions, to an extracranial body target with doses at least biologically equivalent to radical course when given over a protracted conventionally fractionated schedule” (6). In particular, SBRT could be considered a therapeutic option for obtaining long-lasting palliation, and if possible, with a curative aim in oligometastatic patients and/or patients with a long life expectancy. To treat spinal cord compression syndrome, there have been attempts to use SBRT, which can be performed based on 3D-CRT or IMRT technique (Figure 2). Furthermore, IGRT implementation is critical for ensuring the precision and accuracy of SBRT.

**Figure 2:**
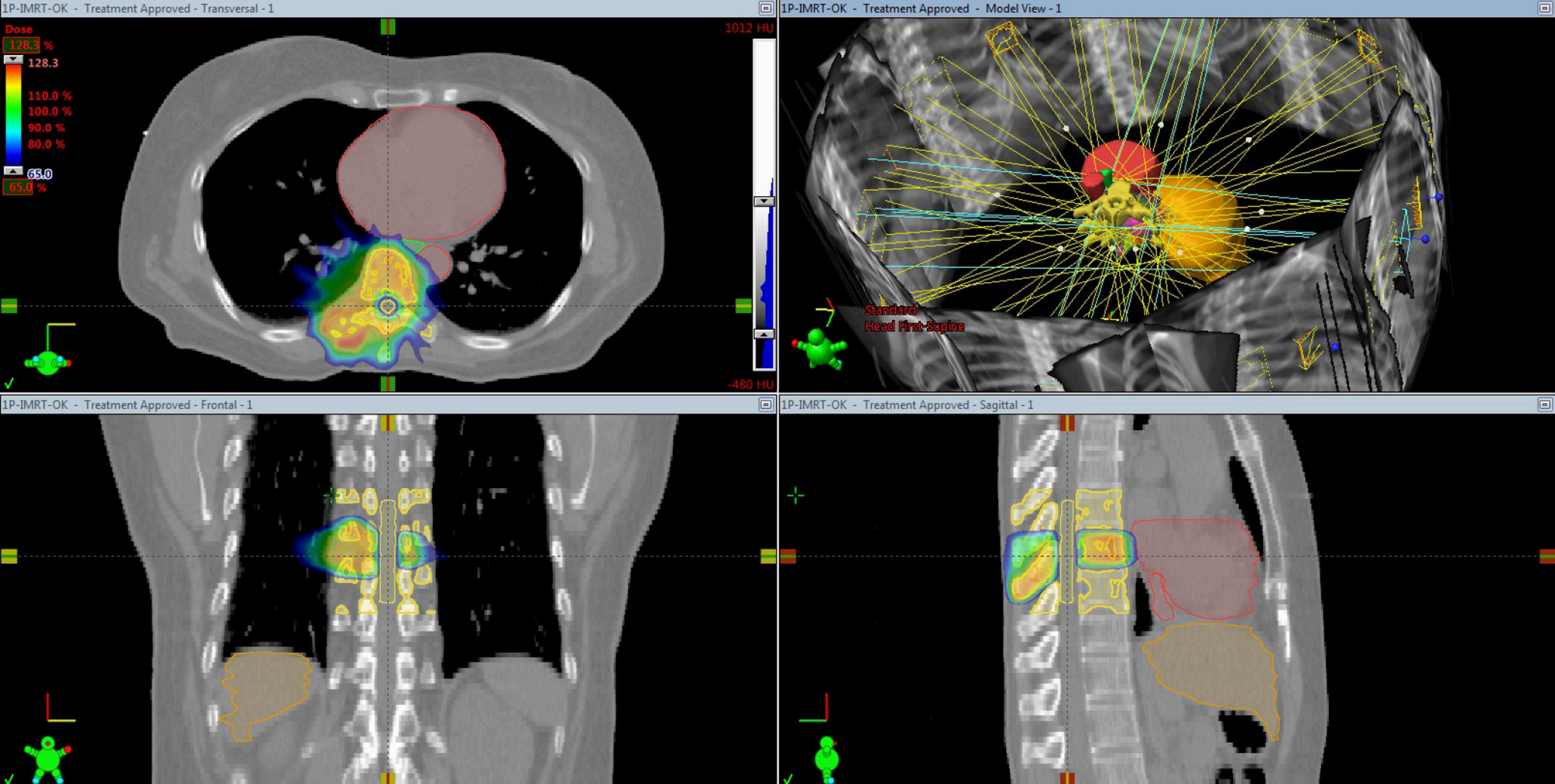
Stereotactic body radiation therapy based on intensity-modulated radiation therapy planning for spine metastasis.

Compared with 3D-CRT, IMRT takes a relatively longer time (several days longer) from the beginning of treatment planning to the start of treatment. The length of time from the RT plan to the start of treatment is considered to be an important factor in the determination of the RT technique because the time to the start of treatment is important in determining the quality of life of patients with spinal cord compression syndrome. From that point of view, the choice of IMRT may have disadvantages. Therefore, in patients with spinal metastases, implementation of IMRT should be performed selectively. In the case of spinal cord compression syndrome, RT is needed as quickly as possible. Therapies such as SBRT or IMRT are not always the best choice, but rather the conventional RT technique, 3D-CRT, may be more appropriate. The actual treatment planning time alone may not be significantly different from that of 3D-CRT (7). However, in order to perform SBRT for spinal cord compression using the IMRT technique, consideration must be given to the additional radiologic imaging, such as spine MRI, needed to facilitate the accurate contouring for the target and normal tissue in tissues such as the spinal cord; additionally, the time for contouring, treatment planning, and quality assurance must be taken into account. In such cases, the difference in time will be greater. Because of this, a delay in treatment initiation of even 1 or 2 days may adversely affect the prognosis of the quality of life for patients with malignant spinal cord compression syndrome. The Canadian Cancer Trials Group is performing a randomized phase 2 study (NCT02512965) to compare spine SBRT of 25 Gy in 2 fractions with conventional RT of 20 Gy in 5 fractions in relation to pain palliation.

It is important to carefully observe changes in the neurological symptoms in patients with bone metastasis, especially in patients with spinal metastasis. An immediate examination and appropriate treatment should be performed when symptoms are present. If RT is needed, based on the overall prognosis of the patient and the extent of the disease, it is important to determine which type of RT should be used. Even when using the latest RT technique, such as IMRT or SBRT, if the appropriate patient selection is not performed, the cost and hospital stay period for the patient may increase without improving the treatment efficiency. Furthermore, many SBRT study protocols do not include tumors within a distance of 3 mm from the spinal cord (8). In addition, it may be better to perform conventional 3D-CRT than to perform SBRT using an IMRT technique. It has also been reported that if the patients are not selected carefully, the risk of disease relapse, especially the occurrence of epidural disease, myelopathy, and vertebral compression fracture, increases (9). Considering this, more sophisticated indications for IMRT for spinal cord compression are needed, and we hope to see this issue resolved in the future. We also expect to see improved RT techniques, including the IMRT technique. This would maintain the existing treatment effect while reducing the treatment planning time compared to the existing 3D-CRT techniques.

The optimal fractionation schedule is determined by tumor factors such as pathology, patient status (e.g., life expectancy and performance status) and other factors such as caregivers' assistance and the cost of treatment. In conventional 3D-CRT, the RT fractionation schedules are used, ranging from a single fraction (e.g., 8 Gy) to fractionated courses (e.g., 30–40 Gy in 10–20 fractions) (10). In SBRT, relatively high radiation doses must be used to achieve optimal outcomes by overcoming tumor resistance. Typically, SBRT is delivered as 18–24 Gy in a single or in two fractions or as 27–40 Gy in 3–5 fractions (11, 12). The Memorial Sloan Kettering Cancer Center is performing randomized phase 3 trials to compare a single fraction SBRT of 24 Gy with a hypofractionated SBRT of 7 Gy to investigate the optimal dose and fractionation regimens relating to local control.

Re-irradiation is an important issue in patients with spinal metastasis. RT can also be repeated using IMRT techniques when the tolerance dose of the spinal cord is limited (13). In particular, the stability and efficacy have been reported not only in retreatment but also in primary care (14). One year after treatment, the local control rate was reported to be approximately 80–85% (15, 16). If there is no response or pain relapse in a previously radiated area, re-irradiation should be considered. The aims of re-irradiation are to achieve pain relief and prevent local complications due to tumor progression without radiation myelopathy. However, because of the low radiation tolerance dose of the spinal cord, retreatment of in-field recurrence by RT is debated. It has been reported that <15% of patients presented with further aggravation of motor function after re-irradiation, while motor function improved in 36% of patients. They suggested a total biological effective dose (BED_total_) <120 Gy_2_ to the spinal cord as a safe dose limit to avoid radiation myelopathy (17).

In conclusion, the appropriate RT technique selection for malignant spinal metastasis is indispensable. For this, an accurate risk assessment through imaging studies and the neurological evaluation of the patient are essential for careful patient selection. Based on these factors, RT strategies should be appropriately decided for each patient while considering the pros and cons of the present RT techniques. Especially in patients with malignant spinal cord compression syndrome, the latest RT techniques, such as SBRT or IMRT, are not always the best choice, but rather the conventional technique, 3D-CRT, may be more appropriate. Further prospective clinical studies and the improvement of RT techniques are needed to better elucidate the role of the latest RT techniques in malignant spinal cord compression syndrome.

## Author Contributions

All authors listed have made a substantial, direct and intellectual contribution to the work, and approved it for publication.

### Conflict of Interest Statement

The authors declare that the research was conducted in the absence of any commercial or financial relationships that could be construed as a potential conflict of interest.
